# Prevalence and Perceived Effectiveness of Pharmaceutical Digital Marketing among Community Pharmacies in Saudi Arabia: A Cross-Sectional Questionnaire-Based Survey

**DOI:** 10.3390/pharmacy8010009

**Published:** 2020-01-15

**Authors:** Younes Ben Said, Nicola Luigi Bragazzi, Natalia Valeryevna Pyatigorskaya

**Affiliations:** 1 Department of Industrial Pharmacy, Sechenov First Moscow State Medical University (Sechenov University), 119991 Moscow, Russia; younisbensaid@gmail.com (Y.B.S.); osipova-mma@list.ru (N.V.P.); 2 Department of Mathematics and Statistics, York University, Toronto, ON M3J 1P3, Canada; 3 Department of Health Sciences (DISSAL), Postgraduate School of Public Health, University of Genoa, 16132 Genoa, Italy

**Keywords:** community pharmacies, pharmaceutical digital marketing, prevalence and effectiveness of promotional tools, pharmacy consumers, pharmaceutical sale representatives

## Abstract

This research analyzes the direct-to-consumer digital marketing technologies in terms of prevalence and effectiveness. A cross-sectional study design was carried out using the non-repeated random sampling technique. Standardized questionnaires were administered by means of face-to-face interviews or online via web software Sphinx (Python Documentation Generator). The relative importance of prevalence (RIP) and the mean evaluation of effectiveness (MEE) were determined for all studied digital media and for all the different groups of respondents (pharmaceutical sales representatives, community pharmacists, consumers, and the entire sample). Inter-individual differences in RIP and MEE were assessed by computing the coefficient of variation, whereas inter-group differences were determined by one-way analysis of variance (ANOVA) with the Scheffé test as a post-hoc test. Research findings showed that, according to the opinion of all respondents, pharmaceutical promotional tools were more prevalent on healthcare websites. However, all respondents considered social media networks and chat messengers to be the most effective in terms of marketing communication. In conclusion, the results of the present research enable a better understanding of which digital platforms are more often used as media for direct-to-consumer pharmaceutical promotion, and which ones are perceived as the most effective for marketing communication.

## 1. Introduction

Nowadays, the increasing popularity of the Internet and social media are pushing pharmaceutical companies toward digital marketing, which has enormous potential value to provide an easy, instantaneous way to gain access to healthcare and pharmaceutical related information for millions of people [1]. The worldwide number of Internet users has been estimated at 1.96 billion [2]. 

From an Arabic perspective, there has been a significant rise in the use of digital resources in the daily life of people [3,4]. A recent report has estimated that in 22 Arab countries, more than 135 million people use the Internet and there are more than 71 million active users of social networks [5]. In Saudi Arabia, the Internet penetration has now reached 89.39%. The active social media users are 23 million, or 68% of the Saudi Arabian population. Mobile penetration stands at 129% of the total population with 43.80 million mobile subscribers. The average time a Saudi spends on social media via any device is 2 hours 50 minutes daily [6]. According to other reports, Saudis are the largest group of active users of social media such as Instagram, Twitter, and Snapchat in the region. YouTube is the most popular social media in Saudi Arabia with 73% (24.71 million) of total social media users, closely followed by Facebook (62%), Instagram (60%), and Twitter (56%). The most popular messaging services are WhatsApp (72%), Snapchat (43%), and FB Messenger (39%). Saudi Arabia leads the world in users of Snapchat and currently has the world’s largest monthly active Snapchat audience with 14.56 million users [6].

Social media and Internet-based tools or platforms enable people not only to generate, share, and exchange information, ideas, and experiences, but also, from a healthcare perspective, digital channels can be used for a variety of reasons including enhancing healthcare education and implementing pharmaceutical promotions [7,8]. According to a report of the Pew Internet & American Life Project, more than 70% of Internet users seek health information online. More and more people are surfing Internet websites or utilizing social media networks to seek information about diseases, medical treatments, and medications [9,10,11]. Social media are frequently used by healthcare professionals for the purpose of professional development [12]. A recent pilot study that investigated the use of social media among health care professionals in Saudi Arabia found that 79% of users sought health-related information on Twitter, with users reporting that social media increased their medical knowledge and literacy [13]. In another study on the effects of digital marketing on physicians, it was found that webinars had a greater influence for changes in clinical practice compared with other digital media [14]. 

The growing presence of pharmaceutical companies in social media is driven by the marketing needs in increasing the public awareness of their products and services and looking for more effective ways of communication with their target audiences. As a result, traditional pharmaceutical promotion is being gradually replaced by digital marketing due to its great cost-effectiveness, being less time-consuming, and favoring easy interactions with customers [15]. 

Previous studies on Internet platforms for healthcare purposes have classified social media into different types including blogs (e.g., WordPress), microblogs (e.g., Twitter), social networking sites (e.g., Facebook), professional networking sites (e.g., LinkedIn), thematic networking sites (e.g., 23andMe), Wikis (e.g., Wikipedia), mashup sites (e.g., HealthMap), media sharing sites (e.g., YouTube) [7], professional social networking communities (e.g., Sermo and Doximity), and others (e.g., virtual reality and gaming environments) [16]. The success of these types of social media platforms is made up of many indicators such as audience reach (e.g., followers and subscribers), page commenting policy, post source (e.g., consumer, pharmaceutical company), post format, and post interactivity (e.g., the number of “Likes”) [17].

Online marketing uses a wide set of promotional tools: search engine optimization (SEO); content marketing, or creating targeted, value-driven branded content in posting and commenting; e-detailing, digitizing sales content and providing it on a mobile device; e-DTCA, or direct-to-consumer advertising on websites, social media, or other digital channels; influencer marketing, or using key leaders; electronic word of mouth (e-WOM); online pharmacies, etc. [17,18,19]. E-marketing tools are implemented through different social media platforms such as healthcare websites, social media networks, communication applications, webinars/webcasts, email, etc. 

However, despite such a varied body of studies, the issue of effectiveness of a certain type of social media for addressing specific types of health problems remains unexplored [20]. 

Digital marketing shows the highest effect on consumers [21,22] and its impact is constantly increasing [14]. However, it was found that disclosing an affiliation with a pharmaceutical company decreased consumers’ trust in the posted information about a medicine [9]. Some other studies have claimed that DTC promoting health products in social media is linked with inappropriate medication use, and may endanger public health [23]. There is an essential need for more research and a better understanding of how the online presence of pharmaceutical companies can affect consumers’ perceptions of promoting health care products. Digital marketing has a proven role in consumer behavior, however, pharmaceutical companies are still not widely employing digital tools due to limited insight about digital marketing techniques [15] and the situation has to be improved [24].

Our literature review has uncovered little information about the most prevalent and effective digital marketing technologies employed in the Saudi pharmaceutical market. Hence, this study aimed to analyze the digital marketing technologies by studying the prevalence of digital promotion on different digital media and examining the effectiveness of different digital resources, according to the opinions of different participants of the promotion process at the retail level of the pharmaceutical market, namely: pharmaceutical sales representatives (PSRs), community pharmacists, and consumers.

The undertaking of this research was significant not only because it filled important gaps in the existing literature, but also because the expected outcomes of this study could be utilized as a way to optimize the promotion of pharmacy goods and could be used by healthcare decision makers for the development of policies for regulating pharmaceutical promotion.

## 2. Methods

The objectives of this research were as follows: to identify the prevalence of digital promotion employed on different digital media and to determine the digital channels where pharmaceutical promotion are the most effective, according to different participants of promotional process: consumers, community pharmacists, and PSRs.

### 2.1. Study Design and Sample

This cross-sectional study was carried out in community pharmacies using the non-repeated random sampling technique. The data were collected by means of questionnaires. The questionnaire was administered either via face-to-face interviews in community pharmacies in Riyadh (involving one of the coauthors, Y.B.S.) or via online software Sphinx (Python Documentation Generator). No differences could be found between the two ways of administering questionnaires. 

### 2.2. The Questionnaire

The questionnaire was developed ad hoc by the authors. It was designed and adapted based on the group of respondents (PSRs, community pharmacists, and pharmacy consumers). The questionnaire consisted of two question subsets. The first set included items formulated to explore the prevalence of digital marketing. Respondents were asked to choose digital media from the proposed list where they could find pharmaceutical promotional information. 

The questions of the second subset were formulated to estimate the effectiveness of the studied digital marketing tools. Respondents were asked to evaluate each of the proposed digital media, according to the effectiveness of marketing communication (which digital platform is more preferable for finding pharmaceutical promotional information). The questionnaires also contained socio-demographic questions. 

### 2.3. Statistical Analysis

Data obtained from the survey were coded and analyzed using the “Statistical Package for Social Sciences” (SPSS for Windows, version 24.0, IBM, Armonk, NY, USA). The relative importance of prevalence (RIP) and the mean evaluation of effectiveness (MEE) for each digital medium was determined for each different group of respondents (PSRs, pharmacists, consumers, and the entire sample). 

In particular, RIP for each digital media in each different group of respondents (PSRs, pharmacists, consumer, and the entire sample) was calculated using the following formula:

(1) RIP=[(Yes×2+No)/(n×2)]×100%

where Yes is the sum of the positive responses; No is the sum of the negative responses; and n is the number of respondents in each group.

Concerning the effectiveness of marketing communication, respondents were asked to evaluate each of the proposed digital media (“This digital media is the most preferable one for finding pharmaceutical promotional information” and “This digital media is the one I prefer to find pharmaceutical information on”) on a scale from one to six points (where one is the worst rating and six is the best rating).

Based on the results obtained, digital media were ranked for the prevalence of marketing tools and their effectiveness. Inter-individual differences in terms of RIP and MEE were assessed by computing the coefficient of variation, whereas inter-group differences were determined by the one-way analysis of variance (ANOVA) and the Scheffé test as the post-hoc test. A *p*-value < 0.05 was considered statistically significant.

## 3. Results

The total number of participants were 790 people (340 community pharmacists; 50 PSRs; 400 pharmacy consumers). Six hundred eighty-eight participants (87.1%) were men and one hundred and two (12.9%) were women. Socio-demographic characteristics of the sample are represented in Table 1.

The questionnaire was administered to respondents in community pharmacies in different districts of Riyadh including the pharmacy located inside the King Saud University premises. The main population of the university campus comprises university staff members, many of which have a scientific degree, and several of them are customers of the pharmacy.

The specificity of medical care in this country is that patients receive free medications in the pharmacies at governmental hospitals. In retail pharmacies, drugs are mainly bought by patients of private clinics, who are mainly from higher social classes, and among them, there may be people with a scientific degree. People with higher education, or a scientific degree, are usually more interested in conducting various studies and respond more actively to requests to participate in them. This may possibly explain why approximately 10% of pharmacy consumers have a degree.

The following types of digital media were analyzed in this study: healthcare websites (e.g., webteb.com, mayoclinic.org), social media networks (e.g., Facebook, YouTube, Snapchat), emails, and webinars/webcasts. 

**Figure 1:**
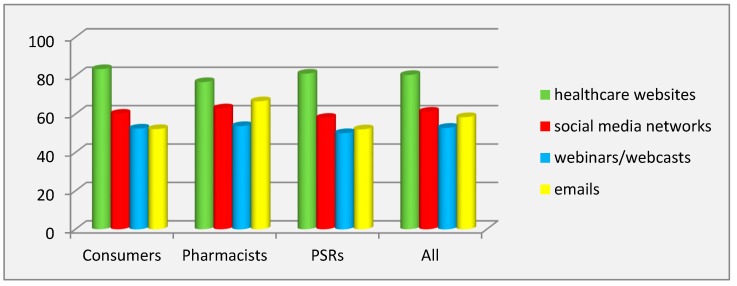
Prevalence of pharmaceutical marketing tools on digital medias.

The study of the prevalence of using digital marketing tools for pharmaceutical promotion revealed that respondents of all groups unanimously considered that promotional tools were most prevalent at healthcare websites: consumers (RIP = 83.38%), PSRs (RIP = 81%), pharmacists (RIP = 76.62%), and all (RIP = 80.32%). Social media marketing (SMM) ranked second for prevalence, according to all respondents (RIP = 61.20%), and to the groups of consumers (RIP = 60.13%) and PSRs (RIP = 58%), while pharmacists considered marketing tools via emails (RIP = 66.62%) more prevalent than SMM (RIP = 62.94%). The third ranked for prevalence was occupied by emails, according to all respondents (RIP = 58.35%), and PSRs (RIP = 52%). The least prevalent, according to all respondents (RIP = 52.78%) and pharmacists (RIP = 53.68%), was pharmaceutical marketing on webinars/webcasts, while according to PSRs (RIP = 50%), promotional tools were not employed on these digital media platforms (Figure 1). 

The calculated values of the coefficient of variation showed significant inter-individual differences (*p* < 0.001) in the group of pharmacists and in the group of all respondents for all of the studied digital media as well as the absence of inter-individual differences for webinars/webcasts in the group of PSRs. ANOVA analysis demonstrated the significant influence of the factor of the respondents’ category on the variation of prevalence for emails (ɳ^2^ = 14.83%), and healthcare websites (ɳ^2^ = 1.79%). The Scheffé test showed the absence of statistically significant differences (*p* < 0.001) between all groups of respondents for all digital medias, except for pharmacists and PSRs for emails.

The study of the effectiveness of using promotional tools on different digital platforms showed that, according to all respondents (MEE = 4.94 ± 1.24 points), consumers (MEE = 4.78 ± 1.34 points), pharmacists (MEE = 5.19 ± 1.19 points), and PSRs (MEE = 5.72 ± 0.45 points), digital marketing was the most effective at social media networks and chats. 

Effectiveness of pharmaceutical promotion on healthcare websites was ranked second unanimously by all respondents with almost identical values of MEE: 4.34 ± 1.27 points (pharmacists), 4.30 ± 1.58 points (PSRs), 4.19 ± 1.28 points (consumers), and 4.25 ± 1.30 points (all respondents). 

The least effective pharmaceutical promotion proved to be emails, according to all respondents (MEE = 2.94 ± 1.44 points), pharmacists (MEE = 3.48 ± 1.39 points), and consumers (MEE = 2.51 ± 1.30 points), whereas PSRs (MEE = 3.42 ± 1.50 points) considered using pharmaceutical marketing on webinars/webcasts as the least effective. 

The values of the coefficient of variation showed the significant inter-individual differences in all groups of respondents for all digital media (22.93% < coefficient of variation < 51.79%), except for social media networks and chats in PSR group (coefficient of variation = 7.88%). ANOVA pointed to the significant influence of the factor of the respondents’ category on the variation of evaluation of the effectiveness of emails (ɳ^2^ = 10.5%, *p* < 0.001), as well as a slightly significant influence on SMM (ɳ^2^ = 3.63%, *p* < 0.001). 

**Figure 2:**
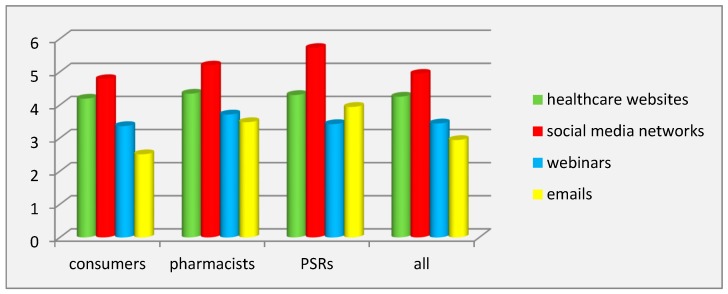
Effectiveness of pharmaceutical marketing tools on digital media.

The Scheffé test revealed significant inter-group differences for the effectiveness of emails between consumers and PSRs (1.44 points) and between consumers and pharmacists (1.20 points) from one another (*p* < 0.001) (Figure 2).

## 4. Discussion

The present cross-sectional questionnaire-based survey assessed the direct-to-consumer digital marketing technologies in terms of the prevalence and effectiveness in Saudi Arabia. The research findings showed that, according to the respondents, pharmaceutical promotional tools were more prevalent on healthcare websites, even though social media networks and chat messengers were considered to be the most effective in terms of marketing communication. 

These findings can be easily explained by the growing popularity of social networks in Saudi society. Today, Saudi Arabia has the largest social media presence in the world. By the end of 2019 in Saudi Arabia, active social media users of YouTube, Instagram, Twitter, WhatsApp, Snapchat and FB Messenger are expected to stand at an incredible percentage (67.95%) of the total population [2]. 

The results of the present research enable a better understanding of which digital platforms are more often used as media for direct-to-consumer pharmaceutical promotion, and which ones are perceived as the most effective for marketing communication.

Our results are in line with the existing scholarly literature. For instance, Liu and Gupta [22] found that the Internet in terms of DTCA expenditures for anti-hyperlipidemia drugs versus conventional channels (like TV) showed significant, positive, and long-term effects on patient behavior (including visits to physicians), even if with a certain degree of variability across consumer sub-groups. 

Similar statements have been reported in the review article on digital pharmaceutical marketing by Dhara et al. [15]. Digital marketing has expanded to become an integral component of our daily life. However, the authors noted that the pharmaceutical industry has not completely exploited the enormous potential of digital marketing, limiting itself to adopting a few tools and channels other than their websites. According to our findings, the effectiveness of pharmaceutical promotion on healthcare websites was ranked only second by all respondents with almost identical values of MEE, and the most effective media for digital marketing appeared to be social media networks and chats, which are emerging tools and devices with an expected high impact on consumer behavior [15]. 

Furthermore, confirming that there is still room for an increased online presence of pharmaceutical marketing, the present research found that digital pharmaceutical marketing utilizing webinars/webcasts was the least prevalent, according to all respondents and pharmacists, with PSRs considering that webinars/webcasts were little used or under-used for direct-to-consumer pharmaceutical promotion. However, in other studies on the effects of digital marketing on physicians, it was found that webinars had a greater influence for changes in clinical practice when compared with other digital media [14]. The effectiveness of webinars may be explained due to the focused nature of the discussion on a specific topic, and the physicians’ engagement with that particular topic.

Moreover, our findings showed that the least effective pharmaceutical promotion proved to be emails. This is in line with the study by Jawaid and Ahmed [14] regarding the impact of pharmaceutical digital marketing on healthcare physicians, in which the authors found that marketing emails have become less popular, probably due to changes in digital marketing that have created a higher rate of competition, with most of the marketing emails finishing into the junk folder or remaining unopened in the inbox.

The findings of our study are consistent with the findings of Robinson et al. [21], who found that drug promotions on the Internet had a strong impact on the population in terms of visits to physicians, requests for specific treatments, and physicians’ prescribing practices. In more detail, the findings of this study revealed that respondents of all groups unanimously considered that pharmaceutical promotion were most prevalent at healthcare websites, even though most physicians had a negative view on DTCA, in that most information displayed was unreliable, fake, misleading, and incomplete, providing insufficient details concerning costs, alternative treatments and management options, and adverse effects. Regulatory issues should be considered to preserve public health and increase medical literacy.

The importance of credibility, as an important variable in the world of digital marketing, has also been explored by Reyes-Menendez et al. [25]. The authors quantitatively assessed the importance of e-WOM for the consumer decision-making process in the tourist sector and the determinants of its adoption on TripAdvisor and other social tourism channels. By means of the elaboration likelihood model (ELM) and the partial least squares structural equation modeling (PLS-SEM) techniques, the authors found that the volume of e-WOM, source credibility, consumer involvement, and perceived e-WOM credibility, but not rate extremism, were drivers of the adoption of e-WOM. However, we have not assessed the importance of credibility and this represents the major limitation of our work. Future research is warranted to address this gap in knowledge. Moreover, future studies could exploit user-generated content and artificial intelligence (AI) techniques including the machine learning approach [26,27] to build predictive models in the field of digital pharmaceutical marketing.

## 5. Conclusions

Nowadays, pharmaceutical companies are becoming the main drivers of introducing digital technologies in the healthcare sector in the search for more effective communication with their target audiences. As a result, digital pharmaceutical marketing is gradually replacing traditional promotions due to its great cost-effectiveness, being less time-consuming, and favoring easy interaction with consumers. Previous studies on pharmaceutical marketing have not identified the most used and most effective digital promotion tools in the retail pharmaceutical market of Saudi Arabia.

This research fills a gap in knowledge by identifying the prevalence of marketing tools used through digital media, and by determining among them the most effective for marketing communication, according to the opinions of the different participants involved in the promotion process: PSRs, community pharmacists, and pharmacy consumers.

The study of the prevalence of using digital marketing tools for direct-to-consumer pharmaceutical promotion revealed that respondents in all groups unanimously considered that promotional tools were most prevalent on healthcare websites. The least prevalent, according to all respondents, was pharmaceutical marketing on webinars/webcasts; moreover PSRs denied using these digital platforms with the absence of inter-individual differences. The study of the effectiveness of digital marketing showed that, according to all respondents, pharmaceutical promotion was the most effective on social media networks and chats. The least effective promotion proved to be via emails, according to all respondents, pharmacists, and consumers, whereas PSRs considered using direct-to-consumer pharmaceutical promotion on webinars/webcasts as the least effective.

The findings of this research underline the growing importance of social media networks as a source of health information, thereby pushing the pharmaceutical industry toward these digital media, and emphasizing the need of creating new digital technologies for patients.

The undertaking of this research was significant not only because it filled important gaps in the existing literature, providing data about digital pharmaceutical marketing in Saudi Arabia, but also because the expected outcomes of this study could be utilized as a way for optimizing the promotion of pharmacy goods and its communication with consumers. Furthermore, the results of the present investigation could be used by healthcare decision makers for the development of policies for regulating pharmaceutical promotion and communication.

However, as also previously mentioned, this study suffers from a number of limitations, which should be properly acknowledged. In this study, we analyzed only direct-to-consumer digital pharmaceutical promotion for over-the-counter medicines and pharmacy goods. To understand the general state of digital pharmaceutical marketing, it seems necessary to analyze the promotion of prescription medications. Another major shortcoming was given by the limited statistical analyses, and the absence of predictive models using AI and advanced analytical techniques helpful to the stakeholders.

## Figures and Tables

**Table 1:** Socio-demographic characteristics: gender, age, job experience, and level of education.

Respondents	N	Gender		Age				Job Experience				Level of Education			
		m	f	<30	30–40	41–60	>60	<1	1–4	5–10	>10	Bachelor	Master	Doctor	Other
Pharmacist	340	340	-	-	-	-	-	27	172	75	66	340	-	-	-
PSRs	50	34	16	-	-	-	-	4	22	24	0	49	1	-	-
Consumers	400	314	86	88	108	144	60	-	-	-	-	152	94	37	117
All	790	688	102	-	-	-	-	-	-	-	-	541	95	37	117
